# Disentangling the stochastic behavior of complex time series

**DOI:** 10.1038/srep35435

**Published:** 2016-10-19

**Authors:** Mehrnaz Anvari, M. Reza Rahimi Tabar, Joachim Peinke, Klaus Lehnertz

**Affiliations:** 1 Institute of Physics and ForWind, Carl von Ossietzky University of Oldenburg, Carl-von-Ossietzky-Straße 9–11, 26111 Oldenburg, Germany; 2 Department of Physics, Sharif University of Technology, Tehran 11155-9161, Iran; 3 Department of Epileptology, University of Bonn, Sigmund-Freud-Straße 25, 53105 Bonn, Germany; 4 Helmholtz-Institute for Radiation and Nuclear Physics, University of Bonn, Nussallee 14–16, 53115 Bonn, Germany; 5 Interdisciplinary Center for Complex Systems, University of Bonn, Brühler Straße 7, 53175 Bonn, Germany

## Abstract

Complex systems involving a large number of degrees of freedom, generally exhibit non-stationary dynamics, which can result in either continuous or discontinuous sample paths of the corresponding time series. The latter sample paths may be caused by discontinuous events – or jumps – with some distributed amplitudes, and disentangling effects caused by such jumps from effects caused by normal diffusion processes is a main problem for a detailed understanding of stochastic dynamics of complex systems. Here we introduce a non-parametric method to address this general problem. By means of a stochastic dynamical jump-diffusion modelling, we separate deterministic drift terms from different stochastic behaviors, namely diffusive and jumpy ones, and show that all of the unknown functions and coefficients of this modelling can be derived directly from measured time series. We demonstrate appli- cability of our method to empirical observations by a data-driven inference of the deterministic drift term and of the diffusive and jumpy behavior in brain dynamics from ten epilepsy patients. Particularly these different stochastic behaviors provide extra information that can be regarded valuable for diagnostic purposes.

Systems under the influence of random forcing or in the presence of non-linear interactions with other systems can behave in a very complex stochastic manner^1^,^2^,^3^,^4^. The analysis of such systems must be based on determining characteristics and strength of fluctuating forces as well as on assessing properties of non-linear interactions. This leads to the problem of retrieving a stochastic dynamical system from measured time series. Addressing the question of how to extract a dynamical system from experimental data with a suitable analysis will provide important information on the properties of the system under consideration^3^.

A widely used non-parametric approach for the modelling of complex dynamical systems employs the conventional Langevin equation that is based on the first- and second-order Kramers-Moyal (KM) coefficients – known as drift and diffusion terms –, and all functions and parameters of this modelling can be found directly from the measured time series^1^,^2^. The Langevin equation generates a *continuous* sample path. However, complex systems generally exhibit non-stationary dynamics that can also result in *discontinuous* sample paths of the corresponding time series, which might challenge the use of the Langevin equation.

There is now growing evidence^5^,^6^,^7^,^8^,^9^ that a continuous-time modelling of time series of complex systems, which exhibit distinct characteristics such as heavy tails and occasionally sudden large jumps, should account for the presence of discontinuous jump components. Indeed, a non-parametric modelling of time series with jumps provides an attractive means of conducting research to gain intuition of such processes. Processes with jumps have been widely used to describe the random evolution of, e.g., neuron dynamics^10^,^11^, of soil moisture dynamics^12^, or of financial figures such as stock prices, market indices, and interest rates^13^. Nevertheless, one of the main problems in the study of *discontinuous* stochastic processes is estimating parameters that represent a jump and the distribution of its size. Another problem is to discriminate between variations caused by a continuous stochastic process and genuine discontinuities in the path of the process when using data sampled at discrete time intervals.

Among the many theoretical models^14^ that have been developed to describe discontinuous components, jump-diffusion modelling has received great attention in the literature, since the non-parametric estimation procedure allows to study potential nonlinearities in the drift, in the diffusion, and in the intensity of the discontinuous jump component^5^. Here we show that a stochastic dynamical jump-diffusion modelling enables us to separate the deterministic drift term as well as different stochastic behaviors, namely diffusive and jumpy behavior. We will demonstrate that all of the unknown functions and coefficients of a dynamical stochastic equation that describe a jump-diffusion process can be derived directly from measured time series.

To illustrate the applicability of our approach, we investigate the stochastic behavior of epileptic brain dynamics by analysing long-lasting multi-channel electroencephalographic recordings from ten epilepsy patients. As was shown previously^15^,^16^, the dynamics of the seizure-generating brain area (epileptic focus)—but not of non-affected brain regions—is characterised by a non-vanishing fourth-order KM coefficient and would thus be assigned to the class of discontinuous processes. Here we relate the higher-order (≥4) KM coefficients to the contribution of an underlying jump process and show that during the seizure-free interval, the dynamics of the epileptic focus can be characterised as a stochastic process with small mean diffusion and small mean jump amplitudes compared to the dynamics of non-affected brain regions.

## Results

### From Langevin to jump-diffusion modelling

By definition, a process *x*(*t*) has continuous sample paths if the following relations for the conditional moments hold for small time increments d*t*^1^:


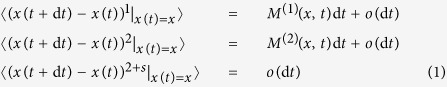


with *s* > 0. Here *o*(d*t*) denotes terms of order higher than linear, which implies that *o*(d*t*)/d*t* vanishes in the limit d*t* → 0. The Langevin-modelling-based KM coefficients are defined as


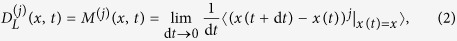


and can be determined directly from measured time series^3^ (we use the subscript L here to distinguish these KM coefficients from the ones that we derive in the jump-diffusion modelling). Note that these KM coefficients^17^ differ by a factor of 1/*j*! from the commonly defined^1^ ones. *M*^(1)^(*x*, *t*) and *M*^(2)^(*x*, *t*) are conditional infinitesimal mean and variance parameters, which are known as the deterministic drift and the diffusion coefficients. In section Methods, we show that processes with non-vanishing and smooth *M*^(1)^(*x*, *t*) and *M*^(2)^(*x*, *t*) but with vanishing higher-order (*j* ≥ 3) KM coefficients generate continuous sample paths.

The probability distribution function *p*(*x*, *t*) for processes with continuous sample paths satisfies a partial differential Fokker-Planck equation, which is of second order in the state variable *x* and of first order in time *t*. There is an equivalence between the Fokker-Planck equation and Langevin dynamics, which means that for a continuous diffusion process the dynamics of *x*(*t*) will be given by a white-noise-driven stochastic equation and has the following expression (using Itô’s interpretation of stochastic integrals^1^,^2^):


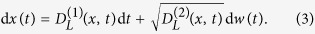


{*w*(*t*), *t* ≥ 0} is a scalar Wiener process and 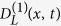 and 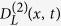 are the drift and diffusion coefficients, respectively. A process *x*(*t*) generated with Eq. (3) is a *continuous* diffusion process for 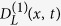 and 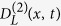 that satisfies the Lipschitz condition. We define the function *f*(*x*) to satisfy a Lipschitz condition on the interval [*a*, *b*] if there exists a constant 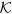 (dependent on both *f* and the interval) such that |*f*(*x*_1_) − *f*(*x*_2_)|≤
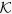
|*x*_1_ − *x*_2_|. Any vanishing KM coefficients of order higher than two, particularly the fourth-order coefficient *M*^(4)^(*x*, *t*), will guarantee that *x*(*t*) is statistically continuous (according to the Pawula theorem a vanishing *M*^(4)^(*x*, *t*) means that all KM coefficients *M*^(*j*)^(*x*, *t*) for *j* ≥ 3 will also vanish)^1^,^18^.

Non-vanishing higher-order (>2) KM coefficients, however, have been observed in various systems^3^,^15^,^19^,^20^,^21^,^22^, which indicates that the corresponding measured time series do not belong to the class of continuous diffusion processes^1^,^2^. In order to improve modelling of such processes, we argue that if all the conditional moments of KM coefficients of order larger than two are non-vanishing, jump events should play a significant role in the underlying stochastic process. We now build a dynamical equation—a jump-diffusion equation—that is able to produce discontinuous sample paths.

A typical jump-diffusion process is given by a dynamical stochastic equation:


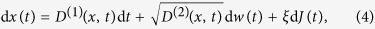


where {*w*(*t*), *t* ≥ 0} is a scalar Wiener process and *J*(*t*) is a time-homogeneous Poisson jump process^23^. This process is characterised by the rate *λ*(*x*, *t*) and the size *ξ*, which we assume to be normally distributed (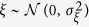). We call 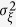 the jump amplitude that may depend on the state variable *x*. Building upon previous works^5^,^17^,^23^, we show below that the drift and diffusion coefficients (*D*^(1)^(*x*, *t*) and *D*^(2)^(*x*, *t*)) of a jump-diffusion process can be related to the conditional moments *M*^(1)^(*x*, *t*) and *M*^(2)^(*x*, *t*). Before doing so, we illustrate the influence of jump events on higher-order conditional moments by calculating the third- and fourth-order conditional moments for the simplest case of a Poisson jump process with a constant jump rate *λ* (see section Methods). We find *M*^(3)^(*x*, *t*) = *M*^(4)^(*x*, *t*) = *λ*. This is the simplest example that provides already an intuitive meaning of higher-order KM coefficients.

Generally, the non-vanishing of the fourth-order KM coefficient could indicate that a jump-diffusion modelling is more appropriate than a Langevin-type modelling, particularly in cases where the corresponding measured time series do not belong to the class of continuous diffusion processes (see section Methods). We now discuss a non-parametric approach to estimate drift, diffusion, and jump characteristics which can be applied to both stationary and non-stationary time series in the presence of discontinuous jump components.

### Non-parametric estimation of jump-diffusion processes

**Theorem.** *A general jump-diffusion process is given by the dynamical stochastic equation* Eq. (4)*, and all of the functions and parameters in this modelling can be found non-parametrically from measured time series by estimating the KM coefficients as:*


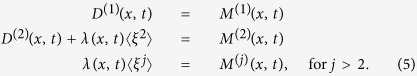


In section Methods, we provide a proof for this theorem. In addition, we derive the conditional moments for bivariate jump-diffusion processes, and its generalisation to higher dimensions is straightforward.

From this theorem, we can derive an equation for the characteristics of the jump process as follows: Using the relation 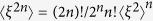 for the Gaussian random variable *ξ* and the last relation in Eq. (5), with *j* = 4 and *j* = 6, we first estimate the jump amplitude 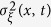 and then the jump rate *λ*(*x*, *t*) as:


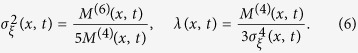


Once the jump characteristics are identified, the second moment *M*^(2)^(*x*, *t*) identifies the diffusion coefficient *D*^(2)^(*x*, *t*) and the first moment gives us the estimate for the drift coefficient *D*^(1)^(*x*, *t*). To estimate the conditional moments *M*^*(j)*^, we can use the Nadaraya-Watson estimator^24^,^25^,^26^, which is a kernel estimator as:


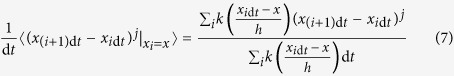


where the kernel *k*(*u*) has the property 
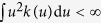
.

### Reconstruction of stochastic processes with jumps

**Figure 1:**
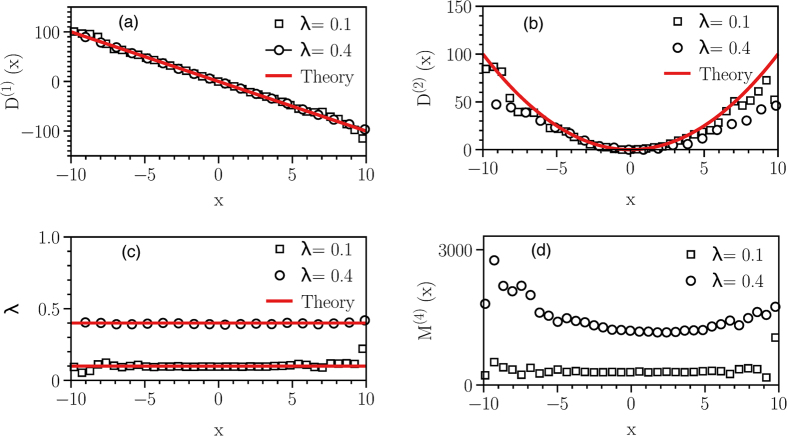
Reconstruction of a Black-Scholes process with jumps. Estimated drift term (**a**), diffusion coefficients (**b**), jump rate (**c**), and fourth-order conditional moment (**d**) for different jump rates using the Nadaraya-Watson estimator with a Gaussian kernel. The time series consisted of 3 · 10^6^ data points, and we here find 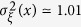. The diffusion coefficient and jump amplitude estimated from normalised time series (with original standard deviation *S*) should multiply to *S*^2^ to get the original diffusion coefficient and jump amplitude. For shorter time series (1 · 10^6^ data points), 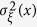 deviates from the expected value by a few percent (not shown).

To demonstrate the validity of our approach, we estimate drift, diffusion, and jump characteristics from time series of well-known jump-diffusion processes with preset coefficients. First, we investigate a Black-Scholes process^27^ in the presence of jumps^28^ (jBSP). In jBSP, we consider a linear drift (*D*^(1)^(*x*) = −10*x*), a quadratic diffusion (*D*^(2)^(*x*) = *x*^2^), a unit jump amplitude (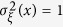), and two constant jump rates (*λ* = 0.1 and *λ* = 0.4). Finite-*N* synthetic data from Eq. (4) may not have jump number 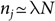. Counting the jumps in each run gives the corresponding correct jump rate for each simulation. We generate synthetic time series by a numerical simulation of the corresponding dynamical equation^29^ using a sampling interval Δ*t* = 0.001. For all estimated functions and coefficients, we obtain a very good agreement with theory (see Fig. 1). The fourth-order moment does not vanish (confirming our approach), and increases with an increasing jump rate *λ*. In addition, we verified that it approaches zero for *λ* → 0. We note that the diffusion coefficient *D*^(2)^(*x*) and the jump characteristics *λ*(*x*) and 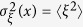 contribute to the second-order KM coefficient (see Eq. (5)). Thus, with a Langevin-type modelling, it is not possible to separate these diffusive from jump contributions. This nonlinear example indicates the importance and physical meanings of higher-order KM coefficients.

As a second example, we investigate an Ornstein-Uhlenbeck process with jumps (jOUP). For *D*^(1)^(*x*) = −*x*, *D*^(2)^(*x*) = 1, unit jump amplitude 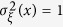, and constant jump rate *λ* = 0.6 we proceed as before, and for all estimated functions and coefficients, we obtain a very good agreement with theory (data not shown).

Given that data sampled at discrete times will always appear as a succession of jumps, even if the underlying path is continuous, we check the robustness of the jump-diffusion reconstruction using different sampling intervals^30^,^31^,^32^,^33^. For both, jBSP and jOUP, all estimated functions and coefficients remain almost unchanged when increasing or decreasing Δ*t* by a factor of 10. Eventually, we turn off the jump process and reconstruct the dynamics in terms of a jump-diffusion process. For all considered sampling intervals, we observe 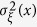 to attain values close to 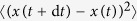 of the generated time series, which can thus be regarded the resolution limit for the jump amplitude.

### Detailing the stochastic behavior of epileptic brain dynamics

Stochastic qualifiers of epileptic brain dynamics that are based on specific characteristics of the first- and second-order KM coefficients estimated using the Langevin-type modelling of electroencephalographic time series can yield valuable information for diagnostic purposes. Previous studies^15^,^16^ have shown that particularly diffusion-coefficient-based qualifiers allow a more detailed characterisation of spatial and temporal aspects of the epileptic process in the affected and the non-affected brain hemisphere. The dynamics of the brain region that is responsible for the generation of focal epileptic seizures (epileptic focus), however, is characterised by a non-vanishing fourth-order KM coefficient, in contrast to the dynamics of other, non-affected brain regions^15^. Thus, pathological brain dynamics appear to not belong to the class of continuous diffusion processes and consequently, the Langevin-type modelling may not capture all aspects of this dynamics^15^. Due to the highly non-linear properties of pathological electroencephalographic time series^34^,^35^, we aim at disentangling the stochastic part of epileptic brain dynamics by explicitly estimating diffusion and jump characteristics.

**Figure 2:**
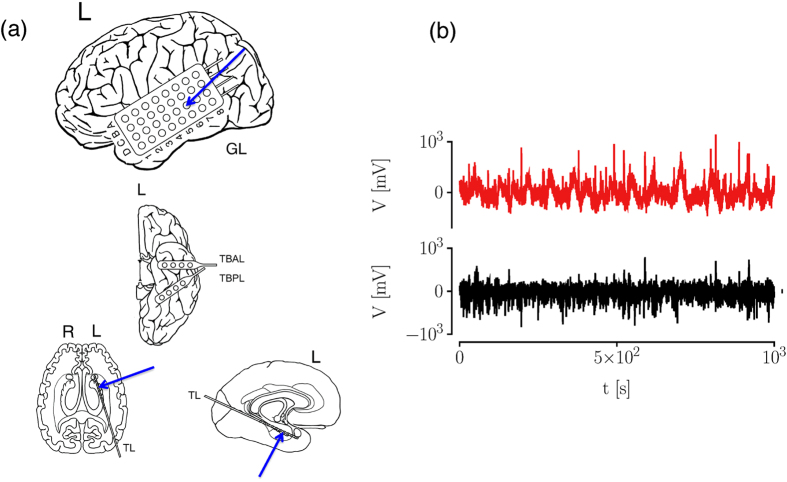
Exemplary recording scheme and intracranial electroencephalographic (iEEG) time series. (**a**) Implantation scheme of intracranial electrodes from a patient with seizures originating in the left mesial temporal lobe: temporal-lateral grid electrode (8 × 4 contacts, GL), two temporal-basal strip electrodes (4 contacts each, TB), and a hippocampal depth electrode (10 contacts, TL; the most anterior contact (TL1) is located ventral to the amygdala and the most posterior contact (TL10) is located within the hippocampus). The latter electrode samples the epileptic focus. (**b**) Segments of iEEG time series recorded during the seizure-free interval from within the epileptic focus (contact TL4, red) and from a distant brain region (contact GLC6, black).

We begin by investigating exemplary brain dynamics from the seizure-free interval of an epilepsy patient. We consider intracranial electroencephalographic (iEEG) time series of 2000 s duration (corresponding to 4 · 10^5^ data points) from within the epileptic focus and from a distant brain region (see Fig. 2). For both time series, we found evidence that our data are Markovian down to the sampling interval employing a least-squares method^3^. Next, we calculate the respective conditional moments of orders 1, 2, 4, and 6, using the Nadaraya-Watson estimator with a Gaussian kernel. For these moments, we obtain finite values in the limit of vanishing time increments, which allows us to conclude that the influence of the measurement noise can be neglected (see section Methods). In the following, we consider the interval *x* ∈ (−200 mV, 200 mV) and report on averaged amplitudes of drift, diffusion, and jump characteristics (thereby we have assumed that the state space of the process is discretized and the conditional average has to be calculated separately for every *x*_*i*_, with binning the state variable into *n*_*b*_ intervals).

**Figure 3:**
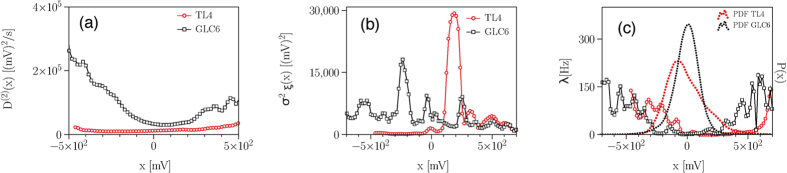
Disentangling stochastic characteristics of epileptic brain dynamics I. Exemplary findings from an epilepsy patient with an epileptic focus in the left mesial temporal lobe. (**a**–**c**) Diffusion coefficients *D*^(2)^(*x*), jump amplitudes 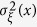, and jump rates *λ*(*x*) together with the respective probability distribution functions *P*(*x*) estimated from normalised iEEG time series (4 · 10^5^ data points) recorded during the seizure-free interval from within the epileptic focus (red, contact TL4) and from a distant brain region (black, contact GLC6; cf. Fig. 2).

The drift coefficients (data not shown) indicate an overall linear damping behavior, however, with small nonlinearities toward larger values of *x* for the dynamics within the epileptic focus^15^. The slopes of drift coefficients differ by about an order of magnitude (within epileptic focus: ≃−0.51 ± 0.05; distant brain region: ≃−3.60 ± 0.12, and from the inverse of the slope of the linear part of the drift coefficients we observe correlation time scales in the order of 1.96 ± 0.20 s for the dynamics within the epileptic focus and of 0.28 ± 0.01 s for the dynamics of the distant brain region. A comparable ratio holds for the averaged amplitudes of the diffusion coefficients, with the one for the former dynamics amounting to about a third of the one seen for the latter dynamics. The diffusion coefficient for the dynamics within the epileptic focus is largely independent of the state variable *x* and for the dynamics of the distant brain region, it depends parabolically on *x* (see Fig. 3a). Overall, these dependences on *x* indicate a multiplicative influence of the noise (cf. Eq. (4)).

From Eq. (5), it is known that the second conditional moment contains contributions from the jumpy dynamics, thus we further disentangle the stochastic part of epileptic brain dynamics and explicitly estimate jump characteristics. We find that the dynamics within the epileptic focus is characterised by a smaller averaged jump amplitude (within epileptic focus: 3400 ± 270 (mV)^2^, distant brain region: 4400 ± 350 (mV)^2^; see Fig. 3b) and by a higher averaged jump rate (within epileptic focus: 18 ± 3 Hz, distant brain region: 8 ± 1 Hz; see Fig. 3c).

**Figure 4:**
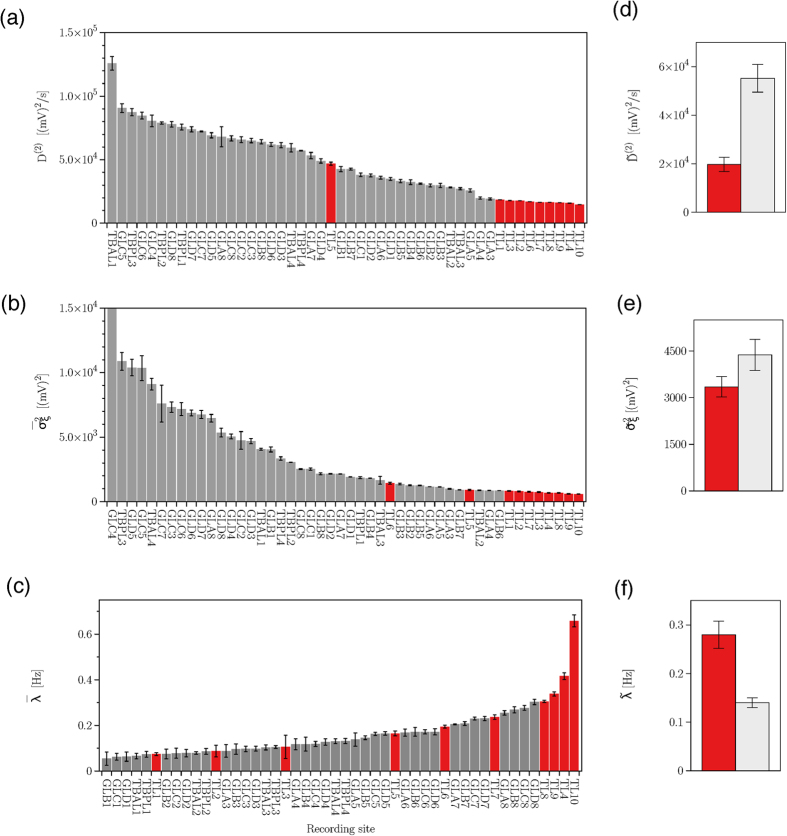
Disentangling stochastic characteristics of epileptic brain dynamics II. Exemplary findings from an epilepsy patient with an epileptic focus in the left mesial temporal lobe. (**a**–**c**) Means and standard deviations of diffusion coefficients (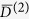), jump amplitudes 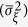, and jump rates (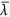) for iEEG time series from all recording sites (cf. Fig. 2). Data from sites within the epileptic focus are colored red. (**d**–**f**) Spatial means and standard deviations of temporally averaged diffusion coefficients 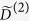. jump amplitudes 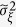, and jump rates 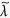 (epileptic focus: red; distant sites: gray).

**Figure 5:**
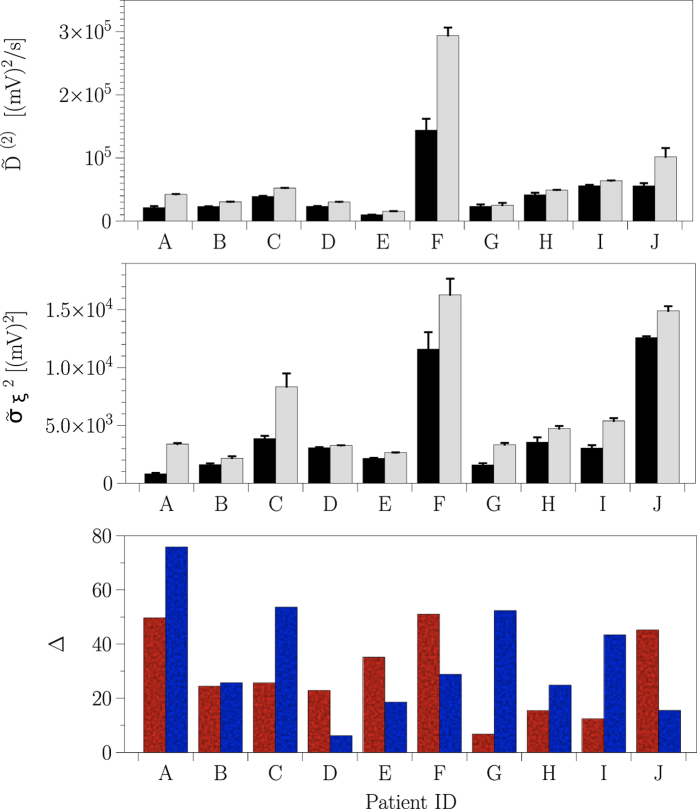
Stochastic qualifiers of brain dynamics for each epilepsy patient. Spatial means and standard deviations of temporally averaged diffusion coefficients 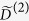 (top) and jump amplitudes 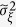 (middle), calculated separately for recordings from within the epileptic focus (black bars) and from distant sites (gray bars). Relative differentiability Δ (bottom) between non-affected (suffix *d*) and affected (suffix *f*) brain dynamics using diffusion coefficients (
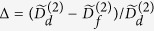
; red bars) and jump amplitudes (
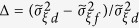
; blue bars).

Next we demonstrate how the aforementioned findings translate to the long-term brain dynamics from all sampled brain regions. To this end, we perform a time-resolved analysis^36^ of the patient’s iEEG time series that were recorded over a period of more than eight days. We subdivide the time series into *n*_*w*_ non-overlapping windows of size 10^5^ data points, calculate estimators 
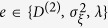
 for each window as outlined above, and by averaging over all windows we obtain their means (denoted as 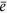) and standard deviations for each recording site (see parts a–c of Fig. 4). Eventually, we derive – separately for the sites from within the epileptic focus and for the distant sites – spatial means and standard deviations of the temporally averaged estimators (see parts d–f of Fig. 4). In general, the dynamics of the epileptic focus in this patient can be characterised by a mean diffusion coefficient 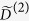 whose amplitude is about half the one seen for the dynamics of the other brain areas. The same holds for the mean jump amplitude 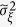, however, the differences between affected and non-affected brain regions are more pronounced, with a mean jump amplitude in the epileptic focus amounting to about a sixth of the one of the other brain areas. The mean jump rate 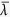 attains high values at some—though not all—recording sites capturing the dynamics of the epileptic focus, and comparably high mean jump rates can also be observed at distant sites. Consequently, differentiability between affected and non-affected brain regions with the mean jump rate 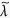 is insignificant. To demonstrate extendability of our observations beyond exemplary data, we now apply the aforementioned steps of a time-resolved analysis for the long-term dynamics of all sampled brain regions from all patients (see section Methods). Figure 5 summarizes our main findings (since differentiability between affected and non-affected brain regions with the mean jump rate 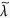 is again insignificant, we omit the display of averaged jump rates). In general, both the mean diffusion coefficient 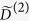 and the mean jump amplitude 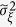 demonstrate a high interindividual variability, both in terms of their sizes and with respect to differentiability between affected and non-affected brain regions. In 5 (out of 10) patients (see Fig. 5c), differentiability with the mean jump amplitude clearly exceeds the one obtained with the mean diffusion coefficient (≈28%), and in 4 patients the opposite holds true (≈21%). In only one case (patient B), both estimators allow for a comparable differentiability (≈22%).

## Conclusion

We presented a method—based on a stochastic dynamical jump-diffusion modelling—that allows one to separate the deterministic drift term as well as different stochastic behaviors, namely diffusive and jumpy behavior. We have argued that when the infinitesimal moments of Kramers-Moyal (KM) coefficients of order larger than two are non-vanishing, jump events should play a significant role in a stochastic process. Indeed, these higher-order KM coefficients carry information about the probability of arrival and about the features of the distribution of the jump size. Our method thus allows one to assign a physical meaning to higher-order KM coefficients in terms of jump rate and jump amplitude. We demonstrated that all of the unknown functions and coefficients of a dynamical stochastic equation that describe a jump-diffusion process can be derived non-parametrically from measured time series, both stationary and non-stationary in the presence of discontinuous jump components.

Through extensive analyses of multi-day, multi-channel electroencephalographic recordings from ten epilepsy patients we demonstrated that the dynamics of the epileptic focus can be characterised as a stochastic process with a smaller mean diffusion coefficient and a smaller mean jump amplitude as compared to the dynamics of distant brain regions. Higher-order KM coefficients thus provide extra information that can be regarded valuable for diagnostic purposes, their relationship to actual physiological/pathophysiological activities, however, would need to be investigated in future studies. Taken together, the findings of our proof-of-concept study underline the high suitability of our generalisation of the Langevin-type modelling to a jump-diffusion modelling to improve the characterisation of pathological brain dynamics beyond a continuous process. We expect that our approach also contributes to a detailed understanding of stochastic dynamics of other complex systems.

## Methods

### Continuity of processes generated by the Langevin equation

In terms of the conditional probability distribution, a continuous process *x*(*t*) satisfies the following continuity condition, given some *δ* > 0^1^


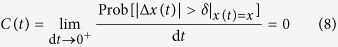


where 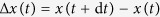. Eq. (8) is sometimes called Lindeberg’s condition^1^. A violation of this continuity condition indicates that the smoothness of the process is not given any more and that discontinuous events like jumps are present in the process.

Here we prove that the processes with non-vanishing and smooth 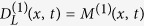 and 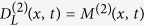 and with vanishing *M*^(*j*)^(*x*, *t*) with *j* ≥ 3 have continuous sample paths. In this case, the Kramers-Moyal expansion for the conditional probability distributions reduces to a Fokker-Planck equation. The short-term propagator *p*(*x*, *t* + d*t*|*x*′, *t*) for the corresponding Fokker-Planck equation is given by^1^,


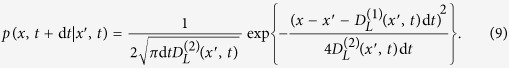


Now we show that the conditional probability distribution function *p*(*x*, *t* + d*t*|*x*′, *t*) satisfies the continuity condition Eq. (8), which means that it describes a continuous stochastic process. Assume that KM coefficients 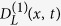 and 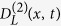 are smooth and not changing dramatically over a short time interval and by substituting *p*(*x*, *t* + d*t*|*x*′, *t*) from Eq. (9) we have,


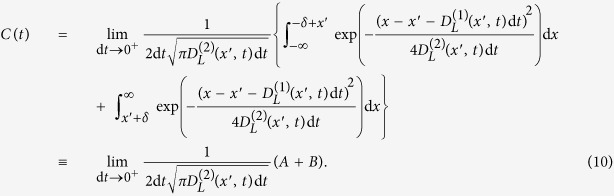


The first term (*A*) in Eq. (10) can be written as:


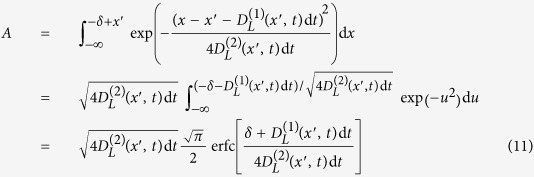


where 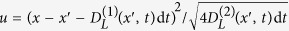. erfc(*x*) can be written in terms of the error function as erfc(*x*) = 1 − erf(*x*), where 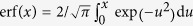. Expanding the expression for *A* in terms of *t* gives,


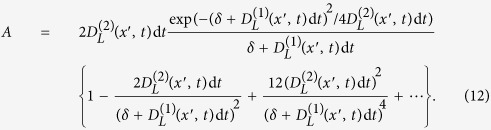


In the limit d_*t*_ → 0^+^ we find that 
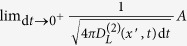
 approaches zero as


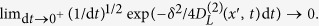


A similar analysis shows that the second term (*B*) in Eq. (10) approaches zero in the limit d*t* → 0^+^. We therefore conclude that the short-time propagator of the Fokker-Planck equation satisfies the continuity condition Eq. (8).

### Non-vanishing higher-order Kramers-Moyal coefficients and the continuity condition

For general processes, the continuity condition (Eq. (8)) can be written in terms of conditional moments *M*^(*j*)^(*x*, *t*) with *j* ≥ 1 and some *δ* > 0 as^37^: 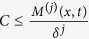. Here we prove the following relation for the continuity condition in terms of Kramers-Moyal coefficients,


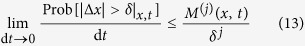


where 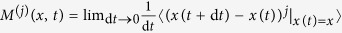. To do so, let us consider the order-*j* conditional moment as,


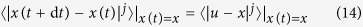


where *x*(*t* + d*t*) = *u*. We can write the conditional moments in terms of a short-time propagator as:


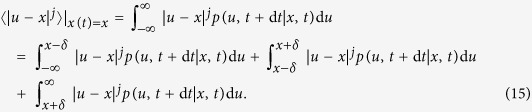


Taking into account the positivity of *p*(*u*, *t* + d*t*|*x*, *t*), one can write the following inequality,


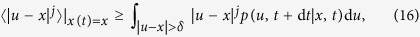


where we have ignored the second term of the r.h.s of Eq. (15). Also using 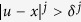 one has:


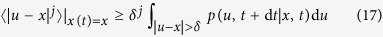


and finally, dividing Eq. (17) by d*t* and in the limit d*t* → 0 we find,


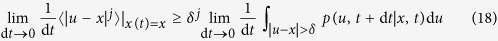


which means that


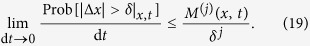


We note that in the moment condition (19) with non-vanishing drift and diffusion coefficients, (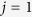 and *j* = 2, respectively) one should consider the case *j* ≥ 3. Therefore any vanishing higher order KM coefficients, particularly the fourth-order coefficient *M*^(4)^(*x*, *t*), guarantee that the process is statistically continuous. Otherwise, the non-vanishing KM coefficients provide an upper limit for the continuity condition. To check the continuity condition for given time series, depending on the problem formulation, the higher-order moment condition may be easier to demonstrate than estimating the tail of the probability distribution.

### Poisson jump process with a constant jump rate

One can imagine that adding jump processes to a diffusion process invalidates the continuity condition Eq. (8). Indeed for such jump-diffusion processes the inequality in Eq. (19) changes to an equality. To illustrate this, we consider—as a simple example—a homogeneous Poisson process, which counts events that occur at a constant jump rate *λ* > 0. This jump rate is the expected number of “events” or “arrivals” that occur per unit time. The number of events in the time interval (*t*, *t* + d*t*] follows a Poisson distribution with associated parameter *λ* d*t*. The jumps have amplitudes 1 and 0 with probabilities *λ* d*t* and 1 − *λ* d*t*, respectively. The probability to observe *k* jumps in the time interval d*t* is given by,


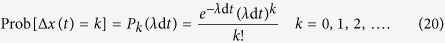


For this process the continuity condition reads,


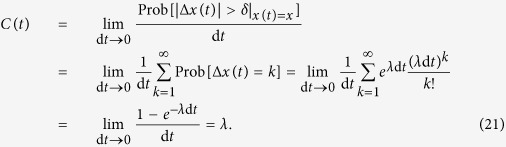


We can also show that all of the conditional moments are equal to jump rate *λ*. For instance, consider the third- and fourth-order KM coefficients as,


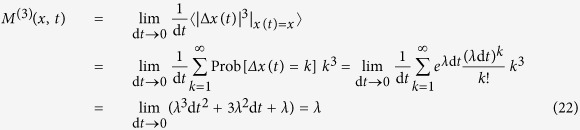


and similarly,


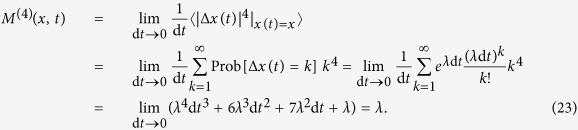


A non-vanishing fourth-order KM coefficient *M*^(4)^(*x*) indicates that (according to the Pawula theorem) for a process with jump events all of the KM coefficients are needed to describe the dynamics of the probability distribution function.

### Proof of relations (5)

To prove the relations in Eq. (5), we can use the different moments of the Wiener process {*w*(*t*), *t* ≥ 0} and the jump process *J*. Using the definition of a Wiener process as the integration of a white-noise signal, we have: 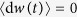, 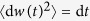 and 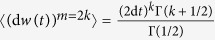. For Poisson-distributed jumps with rate *λ*, the moments of d*J* can be found from its generating function as:


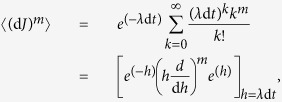


for 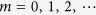. Then one finds


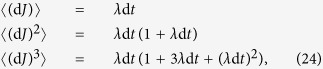


etc. In the limit d*t* → 0 all of the moments are proportional to *λ* d*t*. Therefore we can use the following relations “in-law” and in the limit of vanishing d*t*, d*w*^2^ ≡ d*t* (with 〈d*w*〉 = 0) and d*w*^*m*^ ≡ 0 for *m* ≥ 3. In a similar way, in this limit we have 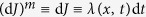 for *m* ≥ 1.

Conditional averaging of Eq. (4) over the Wiener and jump processes gives:


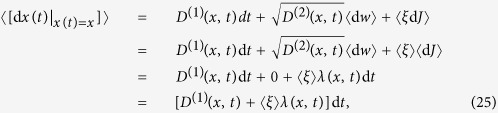


noting to the fact that 〈*ξ*〉 = 0, this proves the first relation in Eq. (5). We also have used the independence of the amplitude of the jumps and of the Poisson process d*J*. Similarly, the second conditioned moment of d*x* leads to:


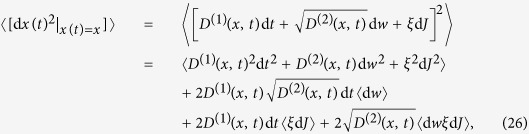


and the terms of order of (d*t*) will be:


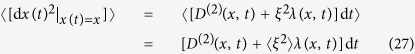


where we used the independence of the Wiener, Poisson and *ξ* processes, i.e., 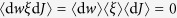. This proves the second relation in Eq. (5). Finally for *k* ≥ 3, we find:


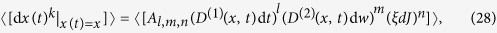


where 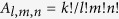, so that *l* + *m* + *n* = *k*. Up to order of d*t* we find:


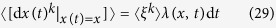


which proves the third relation in Eq. (5).

### Conditional moments of bivariate jump-diffusion processes

Consider bivariate jump-diffusion processes which are given by the following dynamical stochastic equations:


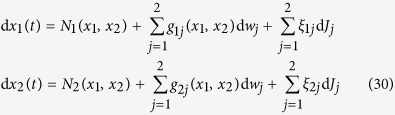


with the normal processes 
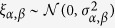
. The Wiener processes {*w*_1,2_(*t*), *t* ≥ 0} are assumed to be independent, and the Poisson-distributed jumps *J*_1,2_ with rates *λ*_1,2_ are uncorrelated. The coefficients *g*_*ij*_ are the diffusion coefficients, and the zero mean amplitudes are assumed to have the correlation 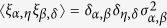.

In this modelling, the two unknown drift coefficients *N*_1_(*x*_1_, *x*_2_) and *N*_2_(*x*_1_, *x*_2_) can be found non-parametrically from measured time series by estimating the first Kramers-Moyal (KM) coefficients as:


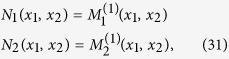


where 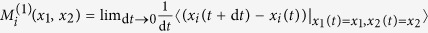. The higher order KM coefficients 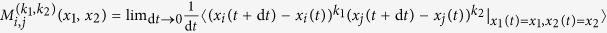 provide a set of the equations for *σ*_*α*,*β*_, *g*_*i*,*j*_, and *λ*_1,2_. For instance, we find:


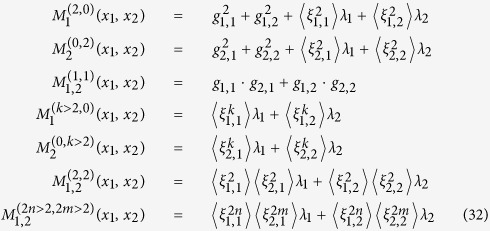


Using the KM-coefficients up to 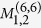, we can find all of the unknown coefficients with a suitable polynomial ansatz for them.

### Influence of measurement noise on Kramers-Moyal coefficients

A measured time series may also contain some other noise, which is not assimilated by the stochastic process. In this case, the time series to be analysed can be written as 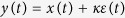, where *x*(*t*) denotes the pure stochastic variable and *ε*(*t*) is an additional noise (with 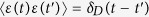 and 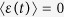; *δ*_*D*_ denotes the Dirac delta function). In general, this type of noise can have its origin in intrinsic components of the complex dynamics or can be caused by an external disturbance, e.g., added to the time series by the measurement process. In the literature, such spoiling noise is called differently, either as observational or measurement noise or as microstructure noise (e.g., in the financial sciences). The conditional moments of the process *y*(*t*) then read^38^:


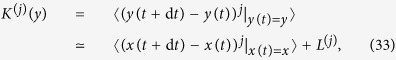


where *L*^(1)^ = 0 and *L*^(2)^ = 2*κ*^2^ and *κ*^2^ is the variance of *ε*(*t*). Note that *κ*^2^ does not depend on d*t*. A finite *L*^(*j*)^ thus causes a strong overestimation of the KM coefficients 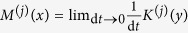 and thus of the jump-diffusion functions (Eq. (6)). It might be worth noting that even in the case of non-negligible measurement noise KM coefficients can be reliably estimated^38^.

For the data shown in Fig. 3, we observed a non-diverging behavior of, for instance 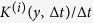 (*i* = 1, 2), which allows us to conclude that the influence of measurement noise can be neglected here.

### Patient characteristics

The ten patients (4 women, 6 men; mean age at onset of epilepsy 14.2 yrs, range 1–33 yrs; mean duration of epilepsy 23.4 yrs, range 1–56 yrs) included in this study suffered from pharmacoresistant focal seizures with different anatomical onset locations, which required prolonged invasive monitoring with intrahippocampal depth electrodes and subdural grid- and strip-electrodes. Decisions regarding electrode placement were purely clinically driven and were made independently of this study. Patients received different antiepileptic drugs (AEDs) with different mechanisms of action, and the majority of patients were under combination therapy with two or more AEDs. During presurgical evaluation AEDs were reduced in a patient-specific manner, and many patients did not have discontinuation of all AEDs. All patients signed informed consent that their clinical data might be used and published for research purposes and are seizure free post-operatively. The study protocol had previously been approved by the ethics committee of the University of Bonn, and methods were carried out in accordance with the approved guidelines.

### Intracranial EEG Recordings

Multi-channel, multi-day intracranial electroencephalographic (iEEG) data were recorded with, on average, 54 electrode contacts (range: 16–88). iEEG signals were band-pass-filtered between 0.1–70 Hz, sampled at 200 Hz (sampling interval Δ*t* = 5 ms) using a 16 bit analog-to-digital converter and referenced against the average signals of two electrode contacts outside the focal region. Reference contacts were chosen individually for each patient. We here consider iEEG recordings from the seizure-free interval with a mean recording duration of 140 h (range: 25–331 h). For our analyses, we divided iEEG data into two groups:

- *epileptic focus*: comprises iEEG data from electrode contacts within the clinically defined epileptic focus;
- *distant*: comprises iEEG data from all other electrode contacts.

## Additional Information

**How to cite this article**: Anvari, M. *et al*. Disentangling the stochastic behavior of complex time series. *Sci. Rep.* **6**, 35435; doi: 10.1038/srep35435 (2016).
